# Effect of pregabalin administration upon reperfusion in a rat model of hyperglycemic stroke: Mechanistic insights associated with high-mobility group box 1

**DOI:** 10.1371/journal.pone.0171147

**Published:** 2017-02-02

**Authors:** Young Song, Ji-Hae Jun, Eun-Jung Shin, Young-Lan Kwak, Jeon-Soo Shin, Jae-Kwang Shim

**Affiliations:** 1 Department of Anesthesiology and Pain Medicine, Yonsei University College of Medicine, Seodaemun-gu, Seoul, Republic of Korea; 2 Anesthesia and Pain Research Institute, Yonsei University College of Medicine, Seodaemun-gu, Seoul, Republic of Korea; 3 Department of Microbiology, Yonsei University College of Medicine, Seodaemun-gu, Seoul, Republic of Korea; 4 Brain Korea 21 PLUS for Medical Science, Yonsei University College of Medicine, Seodaemun-gu, Seoul, Republic of Korea; 5 Severance Biomedical Science Institute and Institute for Immunology and Immunological Diseases, Yonsei University College of Medicine, Seodaemun-gu, Seoul, Republic of Korea; National University of Singapore, SINGAPORE

## Abstract

Hyperglycemia, which reduces the efficacy of treatments and worsens clinical outcomes, is common in stroke. Ability of pregabalin to reduce neuroexcitotoxicity may provide protection against stroke, even under hyperglycemia. We investigated its protective effect against hyperglycemic stroke and its possible molecular mechanisms. Male Wistar rats administered dextrose to cause hyperglycemia, underwent middle cerebral artery occlusion for 1 h and subsequent reperfusion. Rats were treated with an intraperitoneal injection of 30 mg/kg pregabalin or an equal amount of normal saline at the onset of reperfusion (n = 16 per group). At 24 h after reperfusion, neurological deficit, infarct volume, and apoptotic cell count were assessed. Western blot analysis was performed to determine protein expression of high-mobility group box 1 (HMGB1), toll-like receptor-4 (TLR-4), phosphorylated nuclear factor-kappa B (p-NF-κB), interleukin-1beta (IL-1β), tumor necrosis factor-alpha (TNF-α), phosphorylated inducible and endothelial nitric oxide synthase (p-iNOS, p-eNOS), Bcl-2, Bax, Cytochrome C, and caspase-3 in the brain. Pregabalin-treated rats showed significantly improved neurological function (31% decrease in score), reduced infarct size (by 33%), fewer apoptotic cells (by 63%), and lower expression levels of HMGB1, TLR4, p-NF-κB, IL-1β, and TNF- α, compared with control rats. Decreased p-iNOS and increased p-eNOS expressions were also observed. Expression of Bax, Cytochrome C, and cleaved caspase-3/caspase3 was significantly downregulated, while Bcl-2 expression was increased by pregabalin treatment. Pregabalin administration upon reperfusion decreased neuronal death and improved neurological function in hyperglycemic stroke rats. Cogent mechanisms would include attenuation of HMGB1/TLR-4-mediated inflammation and favorable modulation of the NOS.

## Introduction

Irrespective of a history of diabetes, approximately 30–40% of patients that present with acute ischemic stroke exhibit hyperglycemia, which is known to exacerbate clinical outcomes [1]. Unfortunately, the application of intensive glycemic control does not improve outcomes leaving clinicians with an additional burden, whilst already being confronted with limited therapeutic options against stroke in general [2, 3].

The adverse influence of acute hyperglycemia has also been confirmed in animal models of middle cerebral artery occlusion (MCAO) [4]. After energy depletion, ischemic injury universally starts with presynaptic neuronal discharge leading to activation of voltage-gated calcium channels (VGCC) and release of excitatory neurotransmitters in the ischemic core [5].

This excitotoxicity is followed by delayed inflammatory responses in the penumbra, with high-mobility group box 1 (HMGB1) recently identified as the key pro-inflammatory molecule linking these two successive events [6]. In the context of acute hyperglycemia, accumulating evidence suggests that intensification of these pathologic processes leads to increased cerebral injury [7–9]. In addition, hyperglycemia has also been shown to abolish the experimentally proven protective effects of certain agents, such as volatile anesthetic, against cerebral ischemia-reperfusion (I-R) injury [10, 11].

The excitotoxicity persists for hours, even after reperfusion, providing an estimated therapeutic window of up to 10–12 hours [12]. Therefore, we hypothesized that therapies aimed at this initial event would successfully ameliorate its downstream complex biochemical events leading to neuronal loss, and retain their protective effects against cerebral I-R injury even in acute hyperglycemic condition.

Pregabalin, a widely used drug for neuropathic pain, robustly binds to the α2-δ subunit of the VGCC reducing Ca^2+^ influx and release of excitotoxic neurotransmitters at presynaptic nerve endings [13]. Pregabalin’s neuroprotective effect has been evaluated in terms of spinal cord injury [14] and cerebral I-R injury induced by deep hypothermic circulatory arrest [15] or normoglycemic MCAO [16] providing promising results. However, evidence regarding its neuroprotective effects and related mechanisms against stroke is lacking in the context of hyperglycemia, which deserves a high priority considering its prevalence and clinical impact on the outcome.

Therefore, the aim of this present study was to investigate the neuroprotective effects of pregabalin in a rat model of hyperglycemic stroke and its related key molecular mechanisms associated with HMGB1.

## Materials and methods

### Animal preparation

All animal procedures were approved by the committee for the Care and Use of Laboratory Animals, Yonsei University College of Medicine, and were performed in accordance with the Guide for the Care and Use of Laboratory Animals published by the US National Institutes of Health. Rats were fasted except for water for 8 h before surgery, and allowed free access to food and water after surgery. All rats received dextrose (1.2 g/kg) 1 h before MCAO via the tail vein. A blood glucose concentration >11.1 mmol/L was considered as hyperglycemia [17]. The blood glucose concentration was determined at baseline, before MCAO, upon reperfusion, and 24 h thereafter.

### MCAO models and study groups

Male Wistar rats (8–10 wk old) weighing 270–300 g were anesthetised with xylazine (Rompun, Vial Korea, 10 mg/kg) and tiletamine/zolazepam (Zoletil 50, Virbac Korea, 30 mg/kg). To minimize potential suffering from the procedure, supplemental analgesia with local lidocaine infiltration was provided if there was sudden movement or changes in vital sign of animals. The tail artery was cannulated to monitor mean arterial pressure (MAP) and collect blood. The heart rate (HR) was monitored by subcutaneous stainless steel electrodes connected to the power lab system (ML845 PowerLab with ML132; AD Instruments, Colorado Springs, CO). The body temperature was continuously monitored and maintained around 37°C using a heating pad.

The experimental MCAO model was generated as previously described by Longa et al. [18]. Briefly, the left common carotid artery (CCA), external carotid artery (ECA), and internal carotid artery (ICA) were exposed through a midline neck incision. The ECA was dissected further distally and coagulated along with the terminal lingual and maxillary artery branches, which were then divided. A 4–0 monofilament suture (Doccol Corporation, MA, USA) with silicone rubber coated-rounded head was inserted through the proximal ECA stump into the ICA. After 60 minutes of occlusion, the suture was withdrawn, and the skin was sutured with an infiltration of 0.5% bupivacaine for analgesia. For sham operations, all procedures were identical except that the suture was not inserted.

**Fig 1:**
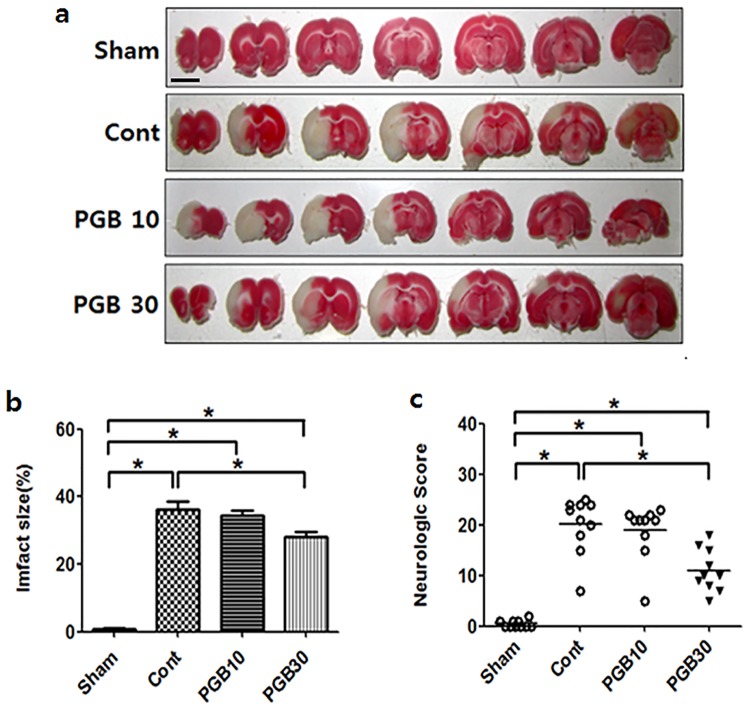
Determination of the dose range for pregabalin treatment. Rats were treated with the indicated dose (mg/kg) of pregabalin (PGB). Representative pictures of TTC-stained brain sections from rat in the different group (a); Infarct volumes expressed as a percentage of area at risk (b); Neurological deficit scores (c). Values are mean ± SD from 8 independent rats. *P <0.05. Scale bar: 1 cm.

The rats were randomly assigned to three groups: 1) Hyperglycemia-Sham group (n = 8), normal saline 2 ml intraperitoneally (IP) after 60 min of ICA exposure without MCAO; 2) Hyperglycemia-untreated MCAO control group (n = 16), normal saline 2 ml IP after 60 min of MCAO, followed by reperfusion, 3) Hyperglycemia-pregabalin treated MCAO group (n = 16), pregabalin 30 mg/kg in normal saline 2 ml IP after 60 min of MCAO, followed by reperfusion. The dose of pregabalin was selected from previous studies of spinal cord injury and deep hypothermic circulatory arrest [14, 15], and also from our preliminary study, in which rats treated with 30 mg/kg demonstrated a smaller infarct size, better neurological function, and lower apoptotic cell count than those treated with 10 mg/kg (Fig 1). The comparative dose of 10 mg/kg was selected from the previous MCAO study in mice under normoglycemia [16]. The dose of 30 mg/kg was chosen for all subsequent experiments. Physical condition of all experimental animals were monitored throughout the procedure and then, every 12 h until the euthanasia was performed with IP injection of an overdose (60mg/kg) of sodium pentobarbital. None of the animals became ill or die prior to the experimental endpoint.

### Neurological deficits

Neurobehavioral assessment was performed assessing four different functions (general status, simple motor deficit, complex motor deficit, and sensory deficit) by combining elements from several previously described scoring systems [19]. The score given in each test (by a researcher blinded to the treatment group) was the sum of all four individual scores: 0 (best)– 48 (worst) score.

### Evaluation of infarct size

At the end of 24 h reperfusion, brains were then perfusion-fixed and 2 mm coronal sections were cut with a tissue cutter. The infarct area of the brain section was determined using 2% 2,3,5-triphenyltetrazolium chloride staining as described previously [16]. The borders of the infarct in each brain slice were outlined and the area quantified using Image J software. For each brain section, the infarct volume was calculated by subtracting the area of the non-infarcted ipsilateral hemisphere from that of the intact contralateral hemisphere. The percentage of infarct volume was determined by dividing the sum for the area of infarction by the total of that of contralateral hemisphere to avoid the influence of edema [20].

### TUNEL assay

The detection of apoptosis on brain paraffin sections was evaluated using the terminal deoxynucleotidyl transferase-mediated dUTP nick-end labeling (TUNEL) staining according to the manufacturer’s instructions (Roche Diagnostics). The number of TUNEL-positive cells was counted in the cortex and the striatum by an observer blinded to the treatment groups using an Olympus microscope at ×400.

### Immunoblot analysis

The tissue samples from the striatum and cortex of the ipsilateral hemisphere were homogenized in a buffer consisting of 20 mM Tris (pH 7.5), 150 mM NaCl, 1% Triton, 1 mM Na2EDTA, 1 mM EGTA, 2.5 mM sodium pyrophosphate, and protease inhibitors. Equal amounts of total protein from each group underwent immunoblotting assays. Proteins were separated on sodium dodecyl sulfate-polyacrylamide gel electrophoresis, and immunoblotting with anti-HMGB1, anti-phospho-inducible nitric oxide synthase (iNOS), anti-iNOS, anti-phospho-endothelial NOS (eNOS), anti-eNOS (Abcam, Cambridge), anti-Toll-like receptor-4 (TLR-4), anti-Actin (Santa Cruz, CA), anti-Bcl-2, anti-Bax, anti-cytochrome C, anti-cleaved caspase-3, anti-caspase-3, anti-phospho-nuclear factor-kappa B (NF-κB), and anti-NF-κB, (all Cell Signaling Technology, Beverly, MA) was performed at least three times each.

### Statistical analysis

All results are expressed as mean (SD). Serum glucose concentrations were analyzed using a repeated-measures analysis of variance (ANOVA). Other variables were analyzed using a two-way ANOVA. The P-values of post hoc tests were adjusted using Bonferroni’s method. Values of P <0.05 were considered statistically significant.

## Results

### Pregabalin treatment conveyed both functional and histological neuroprotection against hyperglycemic MCAO/reperfusion

**Fig 2:**
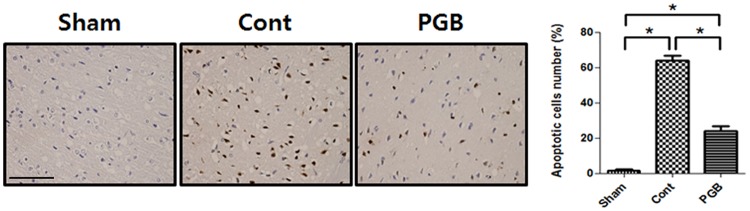
Effect of pregabalin treatment upon reperfusion on apoptosis in the brain of hyperglycemic stroke rat. Pregabalin reduced the number of TUNEL-positive cells compared with the control group. Quantitative representation of TUNEL-positive cells was determined by random counting of four fields per section. Values are mean ± SD from 8 independent rats. *P <0.05. Scale bar: 50 μm.

Dextrose administration increased blood glucose concentrations >11.1 mmol/L until 24 h after reperfusion in all rats without any intergroup differences (S1 Fig). Hemodynamic data, including MAP and HR, also showed no intergroup differences (data not shown). No infarctions were observed in the sham group, while extensive infarct lesions developed in the striatum and lateral cortex of the control group. Compared with the control group, the infarct volume was significantly reduced in all rats in the pregabalin (30 mg/kg) group [38.4 ± 5.5% vs. 25.9 ± 3.5%, P <0.05] (Fig 1a and 1b). The neurologic deficit score of the pregabalin group was significantly lower (better) compared with the control group [11 ± 3 vs. 16 ± 3, P <0.05] (Fig 1c). There were more TUNEL-positive apoptotic cells in the penumbra cortex of the control group than in the pregabalin group [63.9 ± 5.6% vs. 23.8 ± 6.1%, P <0.05] (Fig 2).

### Pregabalin treatment attenuated expressions of HMGB1/TLR-4/p-NF-κB and pro-inflammatory cytokines in the brain tissue

**Fig 3:**
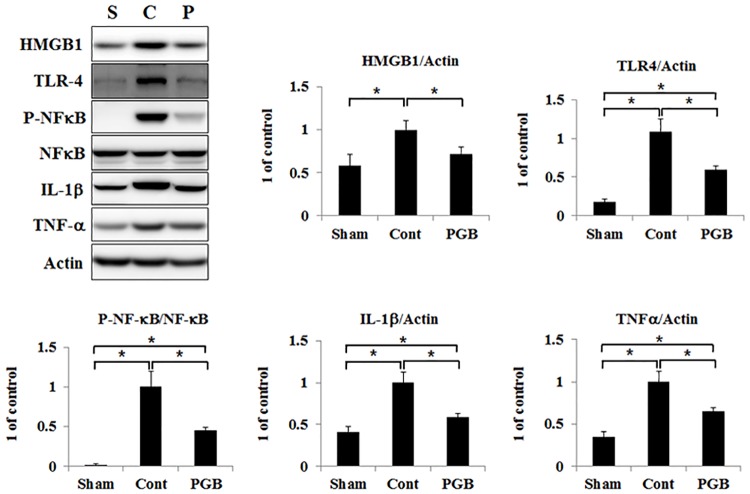
Pregabalin treatment attenuated expressions of HMGB1/TLR-4/p-NF-κB/IL-1β and TNF-α in the brain of hyperglycemic stroke rat. The protein levels of HMGB1, TLR-4, P-NF-kB, NF-kB, IL-1β and TNF-ɑ were detected in the ipsilateral hemisphere of rat brain in each group using western blot analysis. Right panels were quantified signal by scanning densitometry. Values are mean ± SD from 5 independent rats. *P <0.05.

The expression level of HMGB1 was comparable between the sham group and the pregabalin group. Compared to that, it was significantly higher in the control group. Expression of TLR-4 was significantly increased in both the control and pregabalin groups compared with that in the sham group, while it was significantly lower in the pregabalin group than in the control group. Expression of p-NF-κB was significantly increased in both the control and pregabalin groups compared with that in the sham group, while it was significantly lower in the pregabalin group than in the control group (Fig 3). Compared with the sham group, expressions of IL-1β and TNF-α were significantly increased both in the control and pregabalin groups. Their expression levels were significantly lower in the pregabalin group than in the control group.

### Pregabalin treatment decreased expression of p-iNOS and retained expression of p-eNOS in the brain tissue

**Fig 4:**
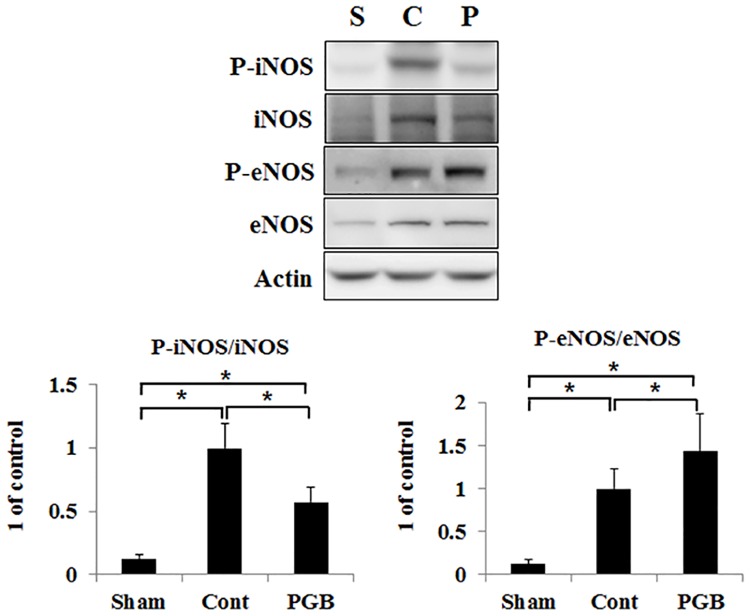
Pregabalin treatment favorably modulated expressions of NOS in the brain of hyperglycemic stroke rat. The western blot analysis performed. The protein levels of P-iNOS and iNOS were detected in the ipsilateral hemisphere of rat brain in each group. Right panel was quantified signal by scanning densitometry. Values are mean ± SD from 5 independent rats. *P <0.05.

The expression of p-iNOS could only be weakly detected in the sham group while it was significantly upregulated both in the control and pregabalin groups compared with that in the sham group. Its expression was significantly blunted in the pregabalin group compared with that in the control group. Levels of p-eNOS were decreased in the control group compared with those in the sham group, while its expression was retained in the pregabalin group (Fig 4).

### Pregabalin treatment favorably modulated expressions of Bcl-2, Bax, cytochrome C, and caspase-3 in the brain tissue

**Fig 5:**
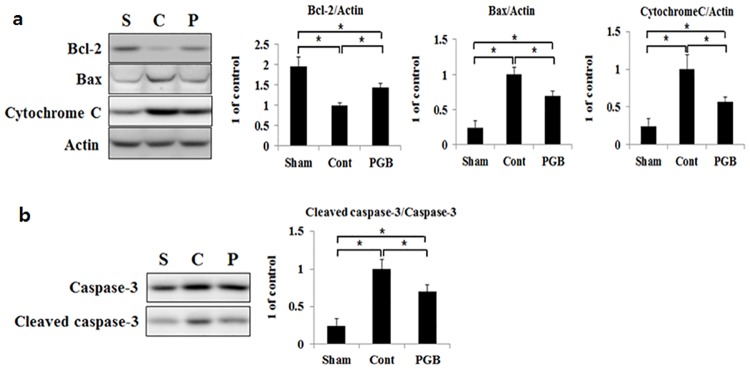
Pregabalin treatment favorably modulated expressions of apoptosis-related molecules in the brain of hyperglycemic stroke rat. The protein levels of Bcl-2, Bax, Cytochrome C (a), Cleaved caspase-3 and Caspase-3 (b) were detected in the ipsilateral hemisphere of rat brain in each group using western blot analysis. Right panels were quantified signal by scanning densitometry. Values are mean ± SD from 5 independent rats. *P <0.05.

Compared with the sham group, both the control and pregabalin groups exhibited a significant reduction in Bcl-2 expression, while it was significantly higher in the pregabalin group than in the control group. On the contrary, expressions of Bax and cytochrome C were significantly increased in both the control and pregabalin groups compared with those in the sham group, while their increases were significantly smaller in the pregabalin group compared with those in the control group (Fig 5a). With regard to cleaved caspase-3, its protein expression level was also significantly increased in both the control and pregabalin groups than in the sham group, while the increase was significantly smaller in the pregabalin group than in the control group (Fig 5b).

## Discussion

In the present study, we observed the functional and histological neuroprotective effects of pregabalin administration upon reperfusion after 1 h of MCAO in a rat model of hyperglycemic stroke. It reduced infarct size by 33%, improved neurological deficits (31% decrease in score), presented with fewer apoptotic cells (by 63%), and demonstrated favorable expression of apoptosis-regulatory molecules in the brain tissue. Activation of HMGB1/TLR-4 signaling and NF-κB-mediated pro-inflammatory cascades was attenuated by pregabalin treatment. Favorable modulation of NOS isoforms was also revealed.

Hyperglycemia has been observed to be present in one-third of all ischemic stroke patients and is known to be associated with poorer prognosis regardless of diabetes history [1]. Of note, a previous meta-analysis reported even higher mortality of stroke in non-diabetic hyperglycemic patients compared with pre-existing diabetic patients [21]. In conjunction, experimental studies have revealed that hyperglycemia exacerbates metabolic deterioration, intensifies the inflammatory response and oxidative stress, augments blood-brain barrier (BBB) disruption, and lowers the availability of NO, leading to increased infarct volume, edema, and hemorrhagic transformation [7, 8, 22, 23]. Even worse, several therapeutic modalities that have shown consistent efficacy in animal studies including the volatile anesthetic post-conditioning lost their neuroprotective effects or even enhanced cerebral damage in hyperglycemic states [10, 11]. The results of intensive glycemic control in an attempt to attenuate exacerbation of stroke have also been largely disappointing [2, 3].

One of the most prominent and initial metabolic issues related to ischemic stroke is distressed Ca^2+^ homeostasis and bursts of excitatory amino acids, which ultimately result in neuronal cell death [5]. Several N-methyl-D-aspartate (NMDA) receptor antagonists and calcium channel blockers (mostly L-type) have shown protective effects against stroke-related damage in a number of experimental models [24]. However, few of them have achieved successful clinical translations mainly because of their adverse psychotomimetic side effects or unwanted influence on the systemic vasculature lowering the perfusion pressure [25, 26]. In consideration of the exaggerated Ca^2+^ imbalance and neuronal excitability [7], hyperglycemic patients may require even higher doses, which may not become applicable on account of these safety issues.

By binding to VGCC, pregabalin suppresses Ca^2+^ influx and has an effect more upstream of the neuronal excitotoxicity, thus blocking the release of not only glutamate but also other excitatory amino acids and providing more comprehensive coverage compared with NMDA antagonists [13]. Indeed, its protective effects have already been demonstrated in experimental models of normoglycemic MCAO and deep hypothermic circulatory arrest with cardiopulmonary bypass [15,16]. Thus, we hypothesized that blocking this initial insult, neuroexcitotoxicity, may ameliorate its downstream events leading to neuronal death and convey neuroprotection against ischemic stroke, even under hyperglycemic condition.

We found in the current hyperglycemic stroke experiment that pregabalin has potent anti-inflammatory properties via downregulation of the HMGB1/TLR-4 signaling pathway and attenuation of p-NF-κB expression. HMGB1, a ubiquitous non-histone nuclear protein, is a key mediator of the immune mechanism in stroke. After extracellular release early after ischemia, it activates immune cells and induces inflammatory cytokine expression via binding to cell surface receptors even hours to days after reperfusion in the ischemic penumbra [6]. Hyperglycemia enhances extracellular release of HMGB1, which has been demonstrated as an important mechanism for worsened ischemic damage [9]. Expressions of its receptors implicated in stroke, including TLRs, have also been demonstrated to be increased by hyperglycemia [27]. Of note, experimental evidences indicate that HMGB1 may participate in further excitotoxicity in the penumbra after stroke by stimulating NMDA receptors and the release of excitatory amino acids [28, 29]. It is likely that pregabalin may have sufficient protective capacity to interrupt such a continuous damaging process in the hyperglycemic states.

One of the most detrimental effects of hyperglycemia in stroke is the more pronounced decrease in blood flow in the penumbra related to NO dysfunction, which may persist late into reperfusion [30]. eNOS activity is critical in maintaining adequate tissue perfusion and BBB integrity under central nervous system (CNS) trauma or inflammation, as well as ischemic stroke [31, 32]. Accordingly, eNOS knockout mice exhibited larger infarction following ischemic stroke [33]. Hyperglycemia has been shown to decrease the activity of eNOS through downregulation of the phosphatidylinositol 3-kinase/Akt pathway [34]. In the present study, pregabalin preserved eNOS activity in hyperglycemic stroke rats, which might also have played a role in its neuroprotective effects.

On the other hand, we observed decreased phosphorylation of iNOS following pregabalin treatment. iNOS has been regarded as a major contributor to the initiation and exacerbation of inflammatory and degenerative conditions of the CNS [35]. It has been associated with superoxide and peroxynitrite generation and nitrosylation of mitochondrial metabolic enzymes, leading to neuronal cell death [35, 36]. The essential molecule for the gene transcription and mRNA stability of iNOS during stroke is NF-κB, which is activated by oxidative stress, glutamate, pro-inflammatory cytokines, and by hyperglycemia per se [37]. Downregulation of iNOS in our hyperglycemic stroke rats by pregabalin might have been attributable to inhibition of the NF-κB transcription pathway, cytokine production, and oxidative stress.

Although complexly interrelated, the overall hierarchy of neuronal injury after oxygen deprivation starts with excitotoxicity and necrosis, which triggers an inflammatory response that causes further neuronal loss by apoptosis [5]. An important downstream mediator of the neuro-excitotoxicity linked with inflammation was found to be HMGB1, which subsequently influences a well-known key inflammatory regulator, NF-κB [6]. In that context, our results provide primary evidence that blocking the foremost and universal event after neuronal energy depletion, excitotoxicity, with pregabalin may improve outcome after ischemic stroke under hyperglycemic conditions. The current study confirms that pregabalin’s beneficial influences are present in all of the above mentioned key inflammatory regulators, NOS, and the anti-apoptotic pathways of neuronal cell death after I-R. An additional strength of the current study is that it firstly addressed the neuroprotective effect of a drug with known clinical safety and reliable pharmacokinetics [38] against hyperglycemic stroke. Considering the high prevalence of hyperglycemia with stroke and its indisputable association with adverse outcome while hindering potentially effective treatments, our results should be of clinical relevance that merits further clinical trials.

This study is subject to the following limitations. First, we cannot draw any conclusion regarding the potential direct influence of pregabalin on each of the specific downstream pathways of neuronal cell death as there are no specific antagonists to pregabalin and it blocks more than just the release of glutamate, including other excitatory neurotransmitters. Second, we did not make any direct comparisons with normoglycemic conditions as they were already reported in a previous study of a mouse MCAO model, which indirectly investigated its calcium-related mechanisms [16]. In that study, although not completely identical in terms of the methods, pregabalin resulted in up to 70% reduction in infarct size as opposed to 33% in our study implicating the adverse influence of hyperglycemia. Moreover, mechanisms for hyperglycemia-induced exacerbation of the stroke pathophysiology including excitotoxicity and HMGB1 cascades have been already well established [7–9, 22, 23]. While avoiding redundancies, we have provided evidence that neuroprotection against stroke can be achieved in the hyperglycemic states in which existing therapeutic modalities have lost their efficacies [39, 40]. Third, although we could clearly delineate pregabalin’s neuroprotective effects at 24 h after reperfusion, delayed neurologic injury may further ensue 24–72 h after reperfusion, which merits a further study.

## Conclusions

Pregabalin administration upon reperfusion conveyed significant functional and histological neuroprotection after 1 h of MCAO under hyperglycemia. These beneficial influences were observed to be associated with major regulators of inflammation, HMGB1, and subsequently, NF-κB and anti-apoptotic pathways, as well as NOS.

## Supporting information

S1 Fig Time course of blood glucose concentrations. MCAO = middle cerebral artery occlusion; PGB = pregabalin group. Values are mmol/L and expressed as mean ± SD. *P <0.05 compared with the baseline in each group.
(TIF)
