# Supplementary figures and images for: Effect of pregabalin administration upon reperfusion in a rat model of hyperglycemic stroke: Mechanistic insights associated with high-mobility group box 1
Source: PLoS One. 2017 Feb 2;12(2):e0171147. doi: 10.1371/journal.pone.0171147 (PMC5289503; doi:10.1371/journal.pone.0171147)

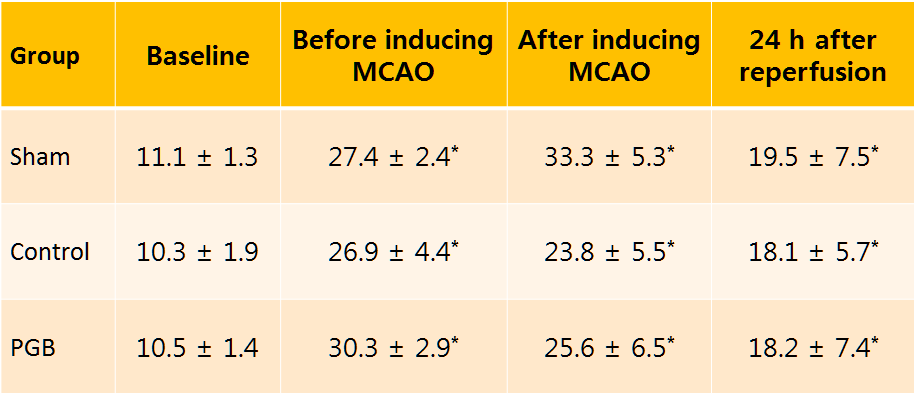

Supplement: S1 Fig — MCAO = middle cerebral artery occlusion; PGB = pregabalin group. Values are mmol/L and expressed as mean ± SD. *P <0.05 compared with the baseline in each group. (TIF) [file pone.0171147.s001.tif]
